# Emergent properties of supramolecular peptide assemblies

**DOI:** 10.1039/d5cc05628d

**Published:** 2025-12-10

**Authors:** Álvaro Vila, Sela González, Ignacio Insua

**Affiliations:** a Centro Singular de Investigación en Química Biolóxica e Materiais Moleculares (CiQUS), Departamento de Farmacoloxía, Farmacia e Tecnoloxía Farmacéutica, Universidade de Santiago de Compostela 15705 Santiago de Compostela Spain ignacio.insua.lopez@usc.es

## Abstract

The self-assembly of supramolecular monomers can change their chemical properties and produce emergent functions that are absent in their dispersed state. In this review article, we describe structural and functional material properties emerging from the self-assembly of peptides, which are based on interactions between neighbouring monomers and the supramolecular environments they create. The non-covalent cooperativity of peptides is here discussed in terms of emergent properties like catalysis, chiral amplification, hierarchical self-assembly and life-like function. These collective effects are rationalised by the monomer packing structure and reactive group proximity, providing a perspective of self-assembling peptide designs and supramolecular material applications, including our own contribution to this topic.

## Abstract 2

The emergent properties of peptides upon self-assembly are discussed in the context of catalysis, structural amplification and life-like function.

## Graphical abstract

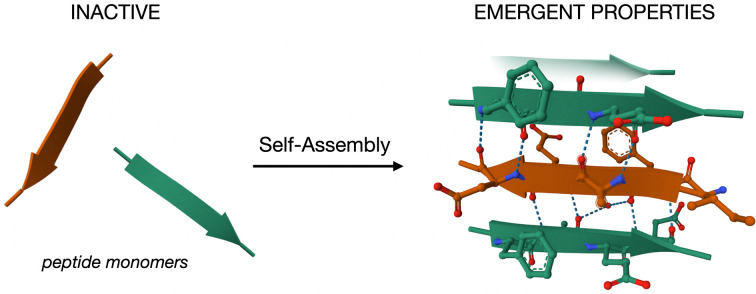

## 1. Introduction

Molecules with complementary geometry and affinity can aggregate in an orderly manner into a wide range of supramolecular structures. This process of self-assembly generates molecular arrays, where neighbouring monomers can influence one another in terms of physical and chemical properties.^1^ In this regard, emergent properties are here defined as the collection of structural and functional effects derived from clustering molecules together, which are absent in their dispersed monomeric state.^2^ These phenomena include fundamental changes in chemical reactivity, like the stabilisation of monomers against hydrolysis in the assembled state^3,4^ or monomer-to-assembly p*K*_a_ shifts,^5,6^ and new collective functions, like catalysis,^7^ luminescence^8^ or motion.^9^ Such emergent properties arise from the supramolecular environments generated by self-assembly, where the conformation of monomers becomes restricted, and their solvation shell is greatly replaced by interactions between bound monomers. For example, the positioning of acidic or basic groups in hydrophobic domains disfavours their ionisation, resulting in a change of p*K*_a_.^10,11^ The close packing of monomers in the assembled state allows structural amplification, where conformational changes in a monomer can be transmitted to its neighbours and ultimately produce macroscopic motility.^12^

The reversible non-covalent interactions that constitute supramolecular materials allow dynamic conversion between assembled and monomeric states,^13^ which can be exploited to switch on and off emergent properties in different ways. Stimuli-responsive monomers can undergo (dis)assembly with light, temperature, pH, redox pairs or enzymes,^14^ allowing their autonomous adaptation to environmental cues. Temporal control over cooperative function can be engineered in dissipative assemblies, which spontaneously decay into dispersed monomers over time.^15^ Conversion between different assembled states (*e.g.* thermodynamic, metastable or kinetically trapped)^16^ can modulate monomer interactions, thus allowing changes in emergent supramolecular behaviour.

**Fig. 1:**
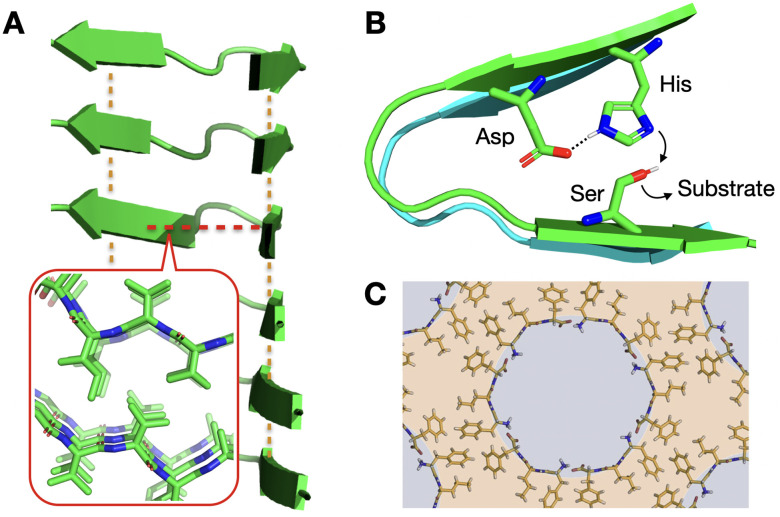
Relevance of supramolecular peptide assemblies with emergent properties. (A) The adoption of secondary structures by peptides, like the β-sheets shown in the image, provides geometrical control over monomer association. In this example, axial elongation is directed by inter-backbone H-bonds (orange lines), while lateral propagation is driven perpendicularly by steric zippers (red line + inset; PDB# 2ONV). (B) Monomer conformation becomes restricted in the assembled state, generating catalytic domains^29^ that are otherwise non-existent in the monomeric (dispersed) state. (C) Heterochiral L–D–L peptide sequences segregate all side chains on one face of their backbone, unlike enantiopure analogues (Fig. 1A inset), allowing the rational design of binding and non-binding surfaces for porous material engineering. Reprinted with permission from ref. 93, copyright 2018, Elsevier Inc.

From the vast range of supramolecular monomers reported, peptides stand out for their versatility and biocompatibility.^17^ The diverse chemical properties of amino acids, which stem from the functional groups on their side chains, allow the modular construction of supramolecular monomers with predictable assembly modes.^18,19^ The adoption of secondary structures by peptides, such as α-helices or β-sheets, can spatially arrange their side chains to provide directional control over self-assembly (Fig. 1A).^20^ Thus, surface domains (*e.g.* hydrophobic, cationic, anionic, *etc.*) can direct monomer packing orthogonally by non-covalent attraction or repulsion, for example, in α/β-barrels^21,22^ and coiled-coil assemblies.^23^ Other important self-assembling motifs beyond the secondary structure, like steric zippers^24,25^ or chain length recognition,^26^ can be exploited to engineer specific packing modes between peptides. As a result, peptides provide a well-established toolbox for the rational design of self-assembling monomers with proven emergent properties.^27^

In this feature article, we put into perspective emergent material properties displayed by peptides, and derivatives thereof in supramolecular assemblies. Three main collective phenomena will be covered in the following sections: catalysis, structural amplification and life-like function. Examples from the last ten years will serve as models to correlate these emergent properties with peptide sequence and supramolecular structure, and are summarised in the final section, including our contribution to the field. While great progress has been made in understanding monomer cooperativity during supramolecular polymerisation,^28^ the study of collective effects post-assembly still requires greater attention to the mechanisms underlying many of the emergent properties observed. We hope this combined discussion of monomer cooperativity post-assembly can guide future developments in functional and adaptive supramolecular materials, both from peptide-based designs and beyond.

## 2. Catalytic assemblies

Given that enzymes are the main biological catalysts, it seems intuitive that peptides, consisting of the same building blocks, can display analogous catalytic behaviour. Although free peptides in solution can show catalytic activity, their organisation in supramolecular structures can dramatically enhance it. This higher activity is explained by the interaction of neighbouring monomers in the assembled state, just like cooperating amino acids in the active sites of natural enzymes.^29^ Thus, bound peptides can affect one another to trigger catalysis, for example, as proton acceptors to activate nucleophiles within the same supramolecular structure (Fig. 1B). Additionally, peptide assemblies can accumulate substrate from the bulk solution, either covalently or by non-covalent interactions, leading to higher local concentrations and hence reactivity in the assembly.

From an evolutionary perspective, peptides formed under prebiotic conditions could have assembled catalytic coacervates, which are considered plausible ancestors of current enzymes.^30^ However, catalytic peptide assemblies lack the structural sophistication of natural enzymes, falling short in replicating their fine substrate specificity and allosteric regulation. In this regard, high substrate affinities and specificities are required in peptide assemblies to mimic the complex metabolic pathways and homeostasis of living organisms. With these exciting challenges ahead, the following sub-sections will summarise recent progress in peptide assemblies catalysing reactions naturally carried out by enzymes. In addition, autocatalysis will be discussed in the context of self-replication, being a current focus of interest in biomimetic self-assembly and bottom-up protocell engineering. Readers interested in detailed catalytic mechanisms and performance (*k*_cat_, *K*_m_) are referred to specialised reviews.^7,31^

### 2.1. Hydrolase activity

Hydrolytic reactions are arguably the most studied reactions in the field of catalytic peptide assemblies, probably due to their biological relevance and ease of monitoring by chromophore production (*e.g. p*-nitrophenol).^32^ As many natural hydrolases use histidine (His) as a proton acceptor/donor in their catalytic centres,^29^ synthetic self-assembling peptides often place His at positions exposed on the surface of the assembly to enable catalysis. For example, His-containing lipopeptides,^33,34^ amyloid sequences^35,36^ and short diphenylalanine monomers^37^ can produce hydrolytic nano-assemblies. Histidine can also be mixed as a free amino acid with peptides for co-assembly into catalytic nanostructures.^38^ In these assemblies, neighbouring monomers cooperate in substrate binding and hydrolysis, justifying the requirement of an assembled state for catalysis. In fact, variations in amino acid order can produce similar assemblies, yet very distinct clusters of neighbouring residues, which affect catalytic performance.^39^ Therefore, catalysis is directly linked to the spatial arrangement of peptide side chains in the supramolecular structure, beyond the overall amino acid composition of the assemblies.

**Fig. 2:**
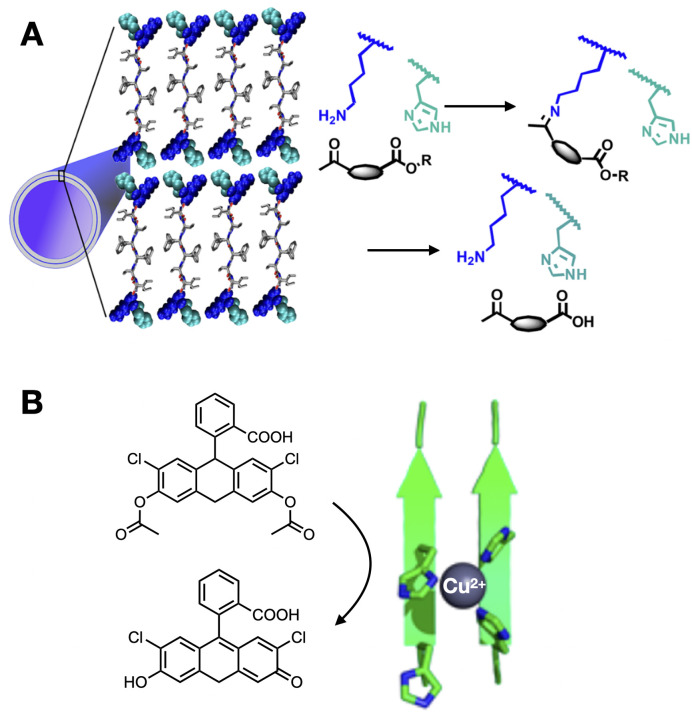
Enzyme-like catalytic function of peptide assemblies. (A) Substrate capture and hydrolysis by peptide nanotubes. Reprinted with permission from ref. 41, copyright 2020, American Chemical Society. (B) Tandem hydrolysis and oxidation reaction catalysed by peptide fibres with Cu^2+^ centres coordinated by His clusters. Reprinted with permission from ref. 48, copyright 2018, American Chemical Society.

The effect of surface groups on the catalytic performance of nano-assemblies was investigated by Das *et al.*, who reported transient nanofibres that spontaneously disassemble based on His-driven self-immolation.^40^ In this design, lipopeptides bearing neutral *p*-nitrophenol esters at the C-terminus are catalytically hydrolysed by adjacent His residues, thus generating anionic repulsion between monomers to trigger supramolecular dissipation. The group later described the cooperative catalysis of histidine and lysine (Lys) residues on the surface of peptide nanotubes, where Lys enhances conversion rates by reversibly capturing substrate molecules on the surface of the assembly as Schiff bases (Fig. 2A).^41^ Further sophistication was achieved by presenting different catalytic surface groups, allowing multistep and convergent cascade reactions on amyloid nanotubes.^42^ Importantly, surface topology can also be exploited for steric patterning, creating grooves on the nanostructures suited to binding specific substrates and thus controlling catalysis by cooperative ligand templation.^43^

The assembly of peptides as coacervates can increase their catalytic activity by the accumulation of the substrate from the medium into these compartments. In addition, coacervation can also promote secondary structure transitions, like β-hairpin folding^44^ and α-helix to β-sheet rearrangement,^45^ resulting in enhanced hydrolysis by these new peptide conformations. Although coacervates are internally disordered aggregates, peptide self-assembly within these droplets can produce ordered catalytic domains, which can be exploited for the challenging hydrolysis and ligation of peptide bonds.^46^

### 2.2. Oxidoreductase activity

Most redox catalysis developed in peptide assemblies employs on-surface bound metals as electron acceptors/donors. For example, and building on the previous section, His clusters in supramolecular nanofibres can coordinate Cu^2+^ as a catalytic cofactor, allowing switching between hydrolase and peroxidase activity in the absence and presence of copper, respectively.^47^ Similarly, amyloid fibres presenting His-Cu^2+^ centres can perform tandem hydrolysis and oxidation cascades exploiting the dual role of His in proton transfer and metal coordination (Fig. 2B).^48^ In an outstanding work by Ding *et al.*, a metal-free peptide assembly with peroxidase activity was developed based on highly ordered His arrays, which cooperate for catalysis by establishing ternary complexes between His domains, H_2_O_2_ and the reducing substrate.^49^

Alternatively, tyrosine (Tyr) can act as an intrinsically redox-active amino acid, able to generate tyrosyl radicals on amyloid nanofibres and drive the oxidative polymerisation of pyrrole.^50^ In this work, the authors rationalise Tyr catalysis by the change in reduction potential upon assembly, demonstrating emergent behaviour in changing the electrochemical properties of this amino acid. While peptides with redox-active pendants (*e.g.* ferrocene and heme groups) have also been exploited as supramolecular catalysts,^32^ these do not rely on monomer cooperation for activity, and hence they have not been included in this discussion.

### 2.3. Ligase activity

Several C–C bond-forming reactions have been catalysed by supramolecular assemblies to connect a range of substrates. Escuder, Miravet *et al.* have investigated self-assembling peptides containing proline (Pro) residues as catalysts for aldol and Mannich condensations.^51^ The authors observed that some Pro-containing lipopeptides were catalytically active only in the assembled state, owing to the hydrophobic environment created by the assembly, which contributes to substrate accumulation and modulates the p*K*_a_ of Pro by cooperative proton transfer across neighbouring prolines.^52^ Interestingly, mixtures of different lipopeptides can produce self-sorted and co-assembled states based on structural complementarity, creating a toolbox to design emergent competitive and cooperative catalytic networks.^53^ Peptide coacervates can also catalyse aldol reactions, where condensed peptide droplets perform a dual catalytic role: in the accumulation of reactants from the dilute phase, and the catalytic action of certain amino acids in crowded, yet highly diffusive, coacervate environments.^54,55^

### 2.4. Autocatalysis

**Fig. 3:**
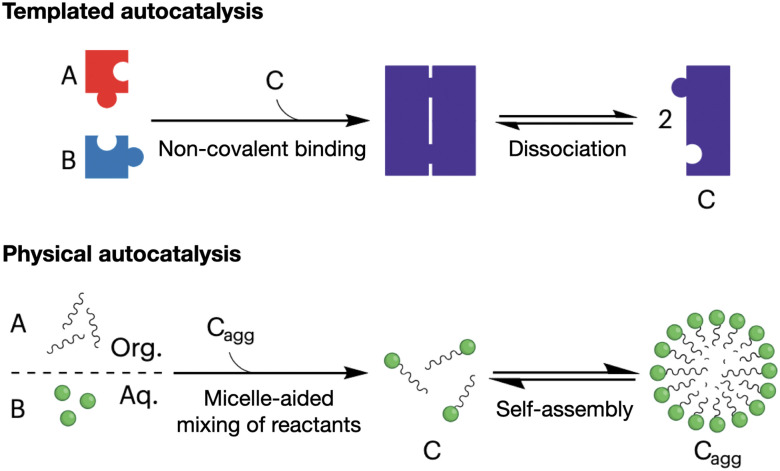
Mechanisms of supramolecular autocatalysis: templated (top) and physical (bottom). Precursor molecules (A and B) react to form the catalytic product (C). '*C*_agg_' denotes the aggregated micellar state of product *C*. Adapted with permission from ref. 58, copyright 2023, Springer Nature.

Autocatalytic reactions are those where the product functions as a catalyst, creating a positive feedback loop that accelerates the reaction as the product concentration increases.^56^ Autocatalytic behaviour is often exemplified by ester hydrolysis reactions, as they produce the corresponding carboxylic acids and a consequent drop in pH, which accelerates further ester hydrolysis.^57^ Alternatively, *supramolecular autocatalysis* is based on the non-covalent association of reactive precursors with their self-assembling product. Fletcher *et al.* classified supramolecular autocatalysis into two types (Fig. 3): (i) in template-driven systems, a product molecule binds the precursors to enhance reactivity by approximating and orienting their reactive groups, and (ii) in physical autocatalysis, a reaction product self-assembles into nanostructures (*e.g.* micelles) that concentrate and favour the mixing of the reactants.^58^ As a result, supramolecular autocatalysis can emerge when covalent reactivity and self-assembly occur in a one-pot fashion, thus coupling monomer synthesis and supramolecular cooperativity.

Regarding templated systems, the first autocatalytic peptide design was reported by Ghadiri *et al.*, who used α-helices with hydrophobic interfaces to bind the precursors and product.^59^ While Ashkenasy *et al.* developed larger trimeric α-helical templates,^60^ the supramolecular propagation of α-helices is mostly limited to small oligomers (*e.g.* coiled-coils, barrels, *etc.*), diverting efforts from the self-assembly community towards peptides able to polymerise as β-sheets. Based on this idea, templated autocatalysis has been observed in different peptide designs capable of assembling into nanofibres.^61–63^ The Otto group has thoroughly studied the autocatalytic self-replication of peptide macrocycles from pools of interconverting oligomers, where molecular selection emerges from the self-assembly of specific macrocycles as nanofibres.^64,65^ Complex behaviour can be engineered in these self-replicating autocatalysts, like competitive precursor specialisation^66^ and parasitism,^67^ mimicking sophisticated relationships between living organisms at the molecular level.^68^

In relation to physical autocatalysis, most published works use fatty acids and other lipid-like derivatives as monomers to produce micelle and vesicle catalysts.^58,69^ In general, these systems are based on immiscible amphiphile precursors, whose mixing and reactivity are enhanced within assemblies of their product. Fletcher *et al.* have exploited dynamic covalent bonds, like those exhibiting disulfide exchange^70^ and alkene metathesis,^71^ to produce transient autocatalytic assemblies with out-of-equilibrium formation-dissipation oscillations. Similar to micelles and vesicles, coacervates can also function as supramolecular nanoreactors with autocatalytic behaviour, as observed both with peptide^72^ and non-peptidic monomers.^73^

**Fig. 4:**
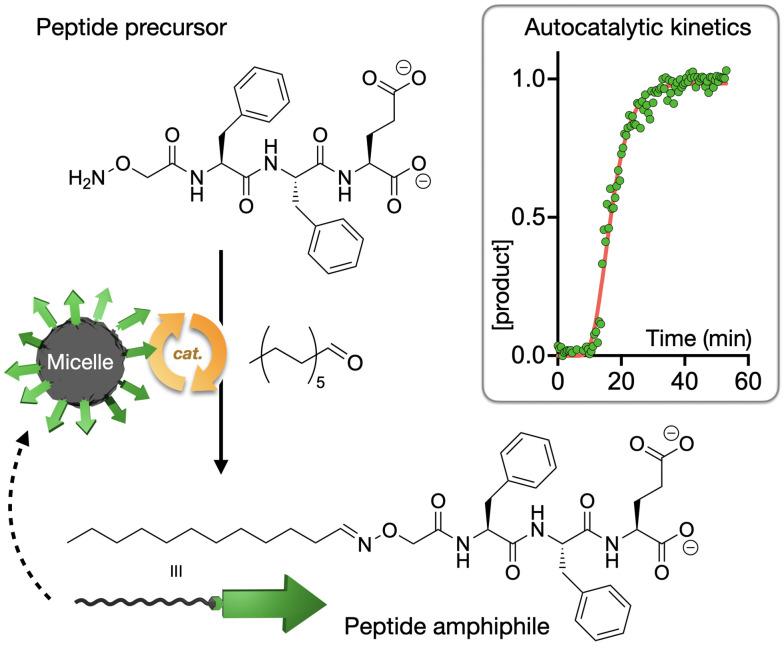
Autocatalytic synthesis of the self-assembling peptide amphiphile dodecyl-F_2_E-OH. The kinetic profile shows normalised product concentration over time with the characteristic sigmoidal shape of autocatalytic processes. Adapted with permission from ref. 74, copyright 2025, Wiley-VCH.

Our group has recently reported the study of autocatalytic peptide amphiphiles (aka lipopeptides), aiming to identify the simplest monomer structure able to display physical autocatalysis.^74^ In this design, peptides bearing reactive hydroxylamine groups condense with aliphatic aldehydes into oxime-connected peptide amphiphiles, which can self-assemble as autocatalytic micelles (Fig. 4). A remarkably short tripeptide amphiphile, dodecyl-F_2_E-OH, was found to be the simplest monomer to display autocatalytic behaviour, establishing a lower limit in the miniaturisation of peptide-based physical autocatalysts in aqueous media. Alternatively, in biphasic systems, a single amino acid lipopeptide was reported by Colomer *et al.* to form autocatalytic micelles in a two-phase water-heptane medium, allowing the competitive selection of products from pools of amino acid precursors.^75^

## 3. Structural amplification

Structural amplification refers to the transmission of monomer geometry to co-assembled neighbours and to the overall supramolecular ensemble, for example, leading to the formation of chiral^76^ and porous^77^ materials. Additionally, the segregation of polar and hydrophobic domains during assembly can amplify the amphiphilic character of a monomer, triggering new assembly modes hierarchically.^78^ In this section, we will discuss several supramolecular effects stemming from the structural amplification of monomer geometry and physicochemical properties. While these concepts have been previously reviewed in isolation, we here provide a holistic view of their supramolecular origin, emphasising monomer packing order in amplifying molecular topology (*e.g.* stereochemistry, binding/non-binding domains and amphiphilicity) in the supramolecular assemblies produced.

### 3.1. Chiral amplification

The chirality of supramolecular assemblies is generally dictated by the stereochemistry of their constituent monomers, which can amplify their geometry to produce torsions in the resulting nanostructures. With all proteinogenic amino acids being chiral, except glycine, the inversion of the D/L configuration of a single residue can dramatically affect self-assembly and structural chirality by introducing conformational mismatches that destabilise secondary structure and steric zipping.^79^ Similarly, changes in D/L amino acid composition can produce changes in the handedness and helical pitch of peptide nanosheets.^80^ While some reports establish a 'C-term rule',^81^ meaning that the overall chirality of an assembly is primarily determined by the D/L configuration of the amino acids at the C-terminus, other works have found that the central core residues of the assembly control supramolecular chirality.^82^ D/L inversions can also modulate the chirality of catalytic nanofibres, resulting in higher yields and enantioselectivity in aldol reactions,^83^ as also observed in other peptide assemblies with helical pitch-dependent catalysis.^84^

**Fig. 5:**
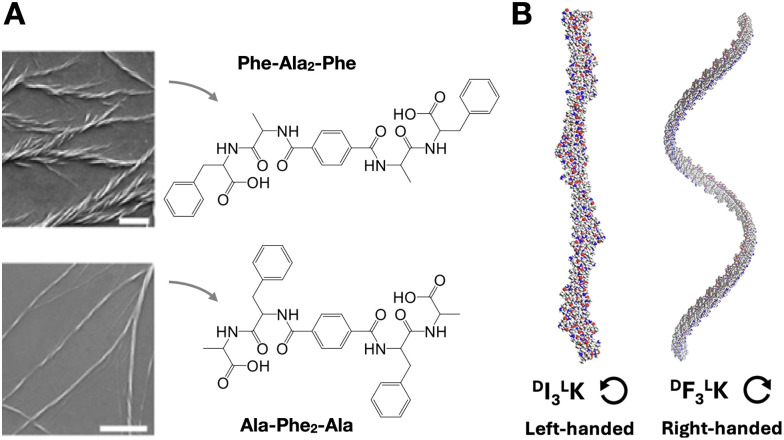
Amplification of monomer chirality into supramolecular helicity. (A) Effect of amino acid order on chiral fibre assembly. Scale bars = 1 µm. Adapted with permission from ref. 82, copyright 2021, Wiley-VCH. (B) Impact of amino acid substitution [*i.e.* isoleucine (I) for phenylalanine (F)] on chiral fibre handedness and helical pitch. Reprinted with permission from ref. 85, copyright 2024, Springer Nature.

Changes in amino acid sequence order can perturb chiral amplification by side chain packing mismatch (*i.e.* unfavoured steric, polar or electrostatic interactions). For example, in tetrapeptides containing phenylalanine (Phe) and alanine (Ala), Phe–Ala_2_–Phe produced larger and wider fibre bundles than the analogue Ala–Phe_2_–Ala, where bulkier Phe residues in the centre of the monomer may hinder fibre elongation (Fig. 5A).^82^ Changes in helical fibre handedness have been observed in peptides maintaining chirality at their alpha carbons, for example between Phe_3_–Lys and Ile_3_–Lys (Fig. 5B).^85^ The distinct side chain geometry and bulkiness of Phe and Ile, with the latter having a second chiral centre at the beta carbon, thus result in opposite M/P helicity from these two peptides. Additionally, the incorporation of non-peptide units at different monomer positions can induce M/P inversions of helical assemblies, for example, using diethylene glycol^86^ or trifluoromethyl^87^ groups.

In co-assembled systems using more than one monomer, chiral amplification can follow the sergeants-and-soldiers principle, where chiral monomers impose spatial organisation over achiral ones, and the majority-rules principle, where the overall chirality of the assembly will be determined by the excess of one of the chiral monomers present.^88^ For example, benzene-1,3,5-tricarboxamide monomers substituted with different amino acids can impose the chirality of their alpha carbon by the sergeant-and-soldiers effect over achiral monomers.^89^ Despite being very interesting and potentially valuable for biomaterial applications, these strategies of chiral amplification remain heavily unexplored in peptides, probably due to the challenge of engineering chiral control in monomers with so many stereocentres.

### 3.2. Packing gaps

**Fig. 6:**
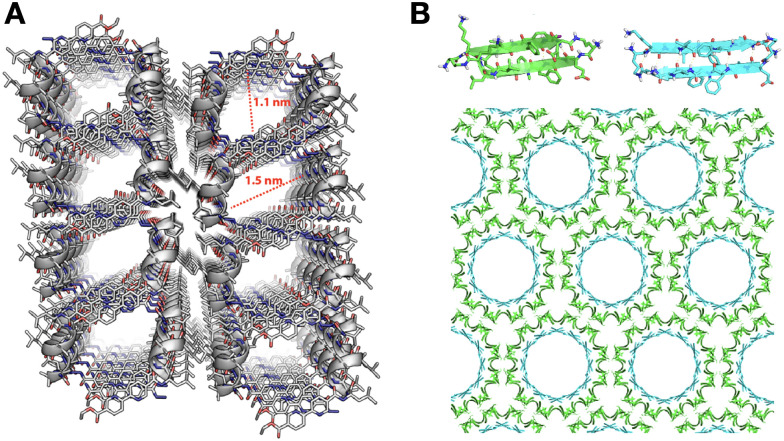
Packing gaps in supramolecular peptide assemblies. (A) α-helical monomers with bipyridine pendants on either terminus for segregated peptide and aromatic assembly. Reprinted with permission from ref. 90, copyright 2022, American Chemical Society. (B) Porous assemblies obtained from two β-sheet-forming peptides (green and blue), consisting of double-walled nanotubes. Adapted with permission from ref. 92, copyright 2017, American Chemical Society.

The packing pattern of peptides can leave gaps in the resulting assemblies, generating porous supramolecular materials by amplification of non-binding monomer domains. While this is an intuitive concept, rational porous material engineering requires fine monomer design considerations, relying on rigid building blocks with binding and non-binding domains in specific orientations. In this regard, porous peptide assemblies have been obtained from helical monomers^90,91^ and β-sheet forming amyloids,^92^ where the secondary structure restricts monomer flexibility and helps present different binding topologies for self-assembly, while leaving non-binding surfaces exposed to the solvent to form the pores (Fig. 6). The size and physicochemical properties of these cavities can be tuned by changes in monomer structure, like sequence extensions or mutations at specific positions, which can be rationally designed to control polarity and potential interactions with guest molecules.^77^ For example, tripeptides with alternating L–D–L chirality segregate all side chains on one face of the peptide backbone to create a hydrophobic binding surface, while leaving the other side of the backbone as an 'empty' non-binding domain to create pores (Fig. 1C).^93^ Additionally, fluorination at phenyl positions of a similar heterochiral dipeptide, ^D^Phe–^L^Phe, affects binding interfaces by incorporation of halogen bonds, thereby modulating packing order and the viscoelastic properties of the material.^94^

### 3.3. Hierarchical self-assembly

**Fig. 7:**
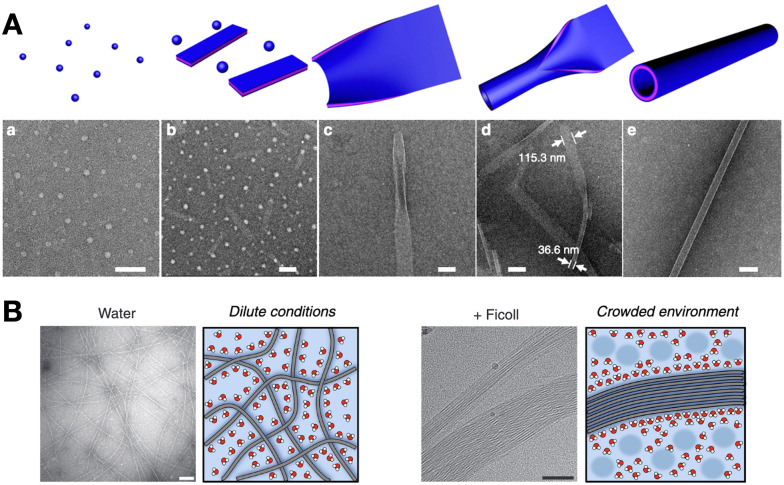
Hierarchical self-assembly examples. (A) Peptoids capable of assembling spherical, flat 2D and hollow nanotube structures progressively (a–e). Scale bars = 200 nm (a, b and d) and 100 nm (c and e). Reprinted with permission from ref. 96, copyright 2018, Springer Nature. (B) Hierarchical bundling of supramolecular fibres in crowded environments, like in the presence of branched polysaccharides (*e.g.* Ficoll). Scale bars = 100 nm. Adapted with permission from ref. 97, copyright 2019, Springer Nature.

The self-assembly of a single monomer can proceed through different packing modes, where one critical assembly state opens access to higher-level elongation stages. This hierarchical mechanism of self-assembly relies on changes in the non-covalent interactions of monomers as they propagate, either amongst themselves or with their medium.^78^ For example, peptide amphiphiles can self-assemble hierarchically (*i.e.* micelles < nanofibres < fibre bundles) with increasing monomer concentration.^95^ In this case, spherical hydrophobic packing dominates at low concentration, then crowded micelles undergo directional elongation by peptide H-bonding into nanofibres, eventually bundling together through their long multivalent surfaces. Peptoids have also been exploited for the hierarchical assembly of nanostructures, sequentially transitioning from particles to sheets and nanotubes by a “rolling-up and closure” mechanism (Fig. 7A).^96^ Alternatively, glycosylated peptides have shown hierarchical assembly pathways, forming individual supramolecular nanofibres under dilute conditions, while bundling together at high concentration due to water exclusion in crowded environments (Fig. 7B).^97^

**Fig. 8:**
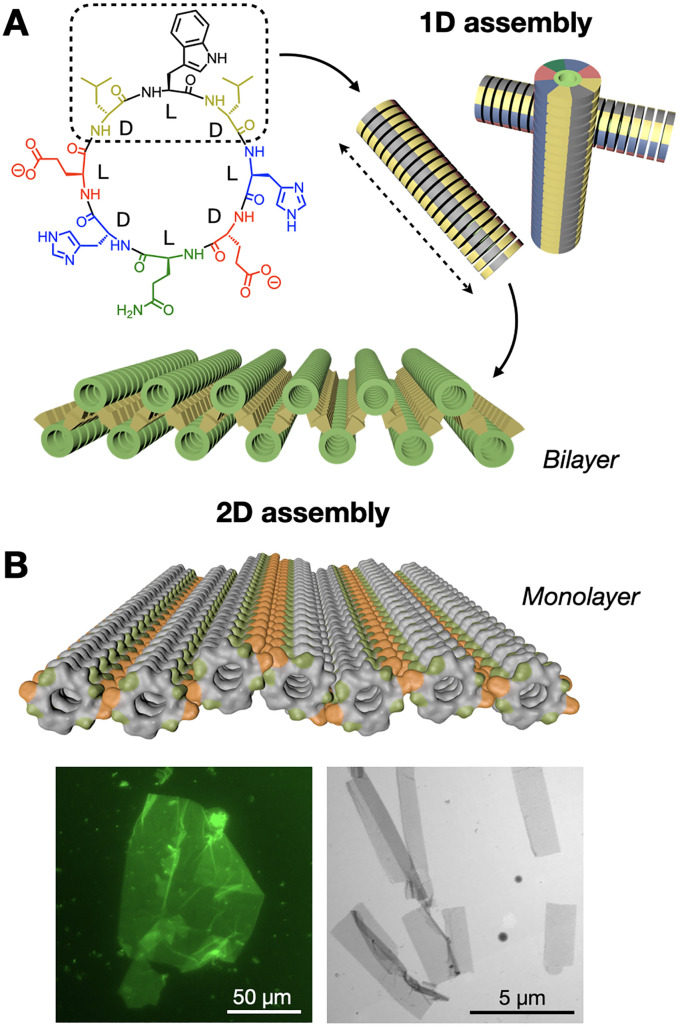
Hierarchical 1D-to-2D self-assembly of cyclic peptides by hydrophobic amplification. (A) The hydrophobic domain of the peptide monomer (dashed square) is amplified along the one-dimensional (1D) nanotubular axis (dashed arrow), triggering the two-dimensional (2D) assembly of bilayered nanosheets. Adapted with permission from ref. 98, copyright 2020, American Chemical Society. (B) Analogous design, now producing nanotubular monolayers by presenting two hydrophobic domains (in orange) positioned on opposite sides of the nanotubes. Epifluorescence (left) and electron (right) micrographs of monolayered 2D nanosheets. Adapted with permission from ref. 100, copyright 2023, Royal Society of Chemistry.

We have developed a new class of hierarchically self-assembling peptides, where monomers can amplify certain topologies during an initial one-dimensional (1D) polymerisation stage, subsequently activating a two-dimensional (2D) assembly mode based on hydrophobic enhancement.^98,99^ Here, cyclic peptides with alternating D/L chirality stack by β-sheet-like H-bonding to produce nanotubes, where the polar and hydrophobic domains of the peptide segregate, creating large hydrophobic nanotube surfaces that further assemble as 2D nanosheets in water (Fig. 8A). Computational simulations demonstrated the stronger solvophobic 2D assembly of 1D nanotubes as they grew longer, consistent with an increase in effective hydrophobic surface as monomers cooperatively array non-polar domains (Fig. 8B).^100^ Importantly, the positioning of hydrophobic domains around the nanotube determined 2D packing geometry, producing nanotube bilayers or monolayers depending on the angle formed by hydrophobic side chains around the peptide macrocycle.

## 4. Life-like function

Understanding the molecular and supramolecular principles of biology remains a central focus of interest in contemporary science towards creating synthetic life.^101^ The bottom-up construction of minimal artificial “cells” (*i.e.* protocells) employs supramolecular building blocks to mimic cellular architectures and functions.^102–104^ In this context, peptides stand out as versatile monomers able to self-assemble as fibres,^105^ coacervates^106^ and vesicles,^107^ to enable the construction of biomimetic systems. Thus, the reproduction of life-like functions by peptide assemblies (*e.g.* confinement in membranes, adaptive fibrillar scaffolds, catalysis, *etc.*) has attracted much attention from the synthetic biology, systems chemistry and prebiotic chemistry communities.^108^ All these life-like functions rely on the collective cooperation of peptide monomers, either as static assemblies with biomimetic behaviour, or dynamically changing supramolecular association to adapt to their environment.

### 4.1. Vesicle and coacervate compartments

**Fig. 9:**
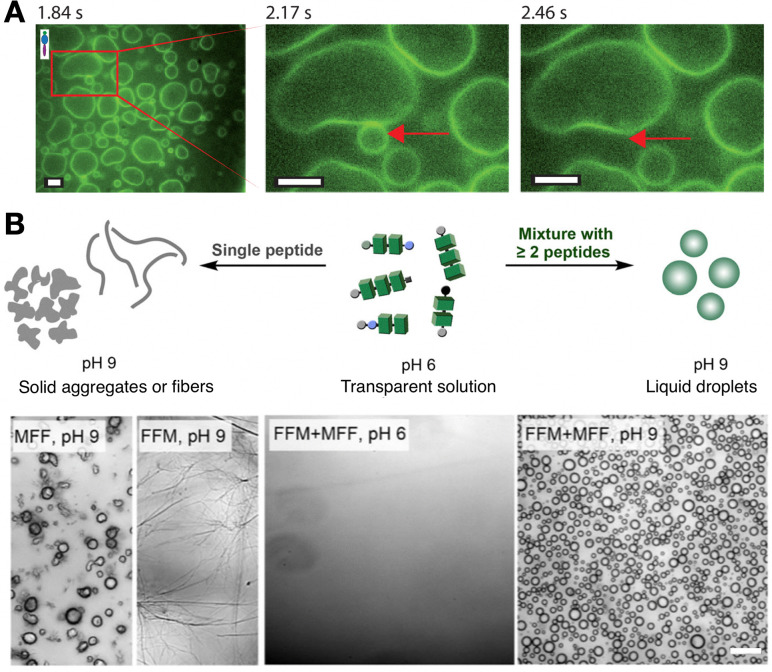
Formation of compartments by self-assembling peptide monomers. (A) Vesicles formed with fluorescently labelled (VPGKG)_40_(VPGIG)_30_ peptide polymer, showing dynamic membrane fusion (red arrow). Scale bars = 5 µm. Reprinted with permission from ref. 111, copyright 2019, American Chemical Society. (B) Co-assembly of tripeptides containing phenylalanine (F) and methionine (M) residues into coacervate droplets. Scale bar = 20 µm. Adapted with permission from ref. 117, copyright 2025, Springer Nature.

The confinement provided by cellular membranes is a defining feature of life, regulating molecular exchange and sustaining essential gradients.^109^ Reproducing such compartmentalisation is a central goal in bottom-up protocell development, enabling the production of minimal cell models to investigate transport, energy transduction and adaptive behaviours.^110^ Schiller *et al.* reported a peptide polymer design, (VPGKG)_40_(VPGIG)_30_, capable of assembling vesicles (Fig. 9A).^111^ In this case, the amphiphilic design of the monomers directs their spatial organisation into bilayered vesicles, where the polarity-based segregation of side chains favours membrane curvature and closure. Das *et al.* recently demonstrated that amphiphilic dipeptides can form dynamic vesicles able to display biomimetic growth, like remodelling and division, outside thermodynamic equilibrium.^112^ Other examples include giant elastin-like peptide polymer vesicles able to encapsulate biochemical reactions that change vesicle morphology,^113^ and short peptide vesicles where inter-monomer affinity determines membrane interaction with the environment.^114^ Altogether, these systems demonstrate that the cooperative interaction between the assembled units enables emergent behaviours, such as growth, division, and adaptive reshaping, that transcend the properties of individual monomers. However, compared to lipid vesicles, peptide-based membranes continue to confront obstacles in achieving long-term stability and selective permeability,^115^ posing exciting challenges for further sophistication of cooperative assembly principles and (supra)molecular designs.

While vesicles establish a membrane barrier between their internal and the external medium, membrane-less coacervates allow free molecular exchange with their environment. Thus, coacervates consist of simple compartments produced by disordered peptide aggregation (*i.e.* liquid–liquid phase separation), able to perform life-like functions as protocells with highly dynamic composition and fluidity.^116^ Caire da Silva *et al.* obtained coacervates by co-assembly of short peptide monomers, which instead produced fibres and solid aggregates in pure samples (Fig. 9B).^117^ The key concept here is the mixing of very similar tripeptides with a single amino acid relocation, Phe_2_Met and MetPhe_2_, leading to attractive interactions while distorting crystallinity by sequence mismatch, thus promoting fluid coacervate aggregation. Indeed, it has been established that methionine (Met) can function as a flexible spacer next to fibre-forming hydrophobic cores (*e.g.* Phe), modulating the structural order and fluidity of the resulting assemblies.^118^

Regarding fuelled out-of-equilibrium designs, Boekhoven *et al.* developed dissipative coacervate droplets formed by RNA–peptide condensation, able to display structural changes such as vacuole formation and fusion.^119^ The group later designed an analogous complex coacervate system, whose sequential formation and decay could produce daughter droplets, which could grow and divide again by consecutive fuelling cycles.^120^ Interestingly, recent evidence that fuelled coacervates do not undergo Ostwald ripening suggests their behaviour can be studied individually, providing a versatile platform to investigate protocellular behaviour and evolution with single droplet resolution and not only in the bulk.^121^ Overall, coacervates consist of crowded compartments without a boundary (*i.e.* membrane) that allow non-specific chemical partitioning and changes in the solvation of internalised molecules, where monomers cooperatively drive covalent and supramolecular processes like catalysis^44,45^ and in-coacervate self-assembly.^122^

### 4.2. Fibrillar scaffolds

Supramolecular peptide fibres have demonstrated great potential in the structural and functional mimicking of cell and tissue behaviour. These assemblies have been studied as synthetic analogues of the extracellular matrix (ECM) and the cytoskeleton, producing fibrous networks that promote natural cell adhesion and differentiation, as well as structural organisation and function in protocells.^105,123^ Stupp *et al.* have extensively investigated peptide amphiphile nanofibres as ECM-like scaffolds, showing that multivalent cell-fibre interactions are critical for biological activity, and ultimately rely on the cooperative presentation of multiple bioactive monomers on the surface of these supramolecular fibres.^124^ Interestingly, the group found that more dynamic and fluid assemblies are beneficial for bioactivity, probably owing to their higher adaptability to rearrange cell-signalling domains on their surface.^125^ Monomer diffusion within the assembly, and hence biological function, could be adjusted by monomer design to pack either in parallel or antiparallel β-sheet configuration.^126^ Similarly, cooperative multivalent interactions on the surface of these assemblies can reversibly control hierarchical inter-fibre organisation as bundles, with direct effects on cell behaviour when used as ECM mimics.^127^ While other groups and peptide designs have successfully achieved ECM-like function and derived applications,^128^ the contribution of the Stupp group to the understanding of supramolecular fibres in terms of monomer cooperativity and dynamics sets a solid foundation to guide future progress in biomaterial-cell interfaces.

**Fig. 10:**
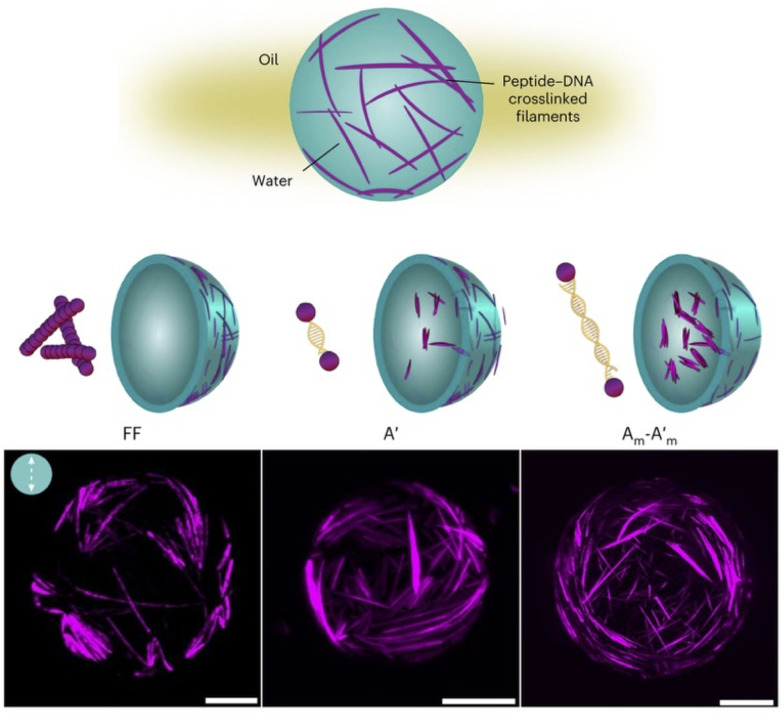
Fibre self-assembly inside water-in-oil emulsion droplets. Dipeptide Fmoc-FF-OH (FF) covalently linked to DNA sequences allowed fibre assembly by FF oligomerisation and controlled fibre cross-linking through complementary DNA hybridisation (*A*′ = 8 base pair DNA; 
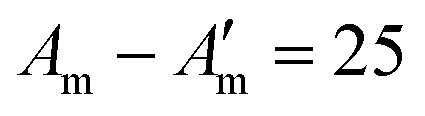
 base pair DNA). The presence and length of the DNA linker affect fibre length and spatial organisation. Scale bars = 20 µm. Reprinted with permission from ref. 132, copyright 2024, Springer Nature.

The ability of peptide assemblies to mimic ECMs can be applied to emulate intracellular fibre networks, like the cytoskeleton, which is responsible for cell structure, motion and organisation.^129^ Despite interesting advances in artificial cytoskeletons for protocell development, like DNA filaments^130^ and diacetylene polymers,^131^ peptide designs remain scarce. One of the few examples was reported by Freeman *et al.*, who developed a hybrid peptide-DNA cytoskeleton mimic encapsulated in water-in-oil droplets; a monomer able to mimic the structure and cross-linking of natural actin microfilaments (Fig. 10).^132^ In this work, short diphenylalanine monomers assembled fibres with pendant DNA strands to interconnect peptide filaments into a dynamic network. The resulting cytoskeleton mimic could adapt its structural organisation and induce morphological changes in the droplets, reproducing essential cellular functions by the cooperative restructuring of supramolecular fibre networks.

**Fig. 11:**
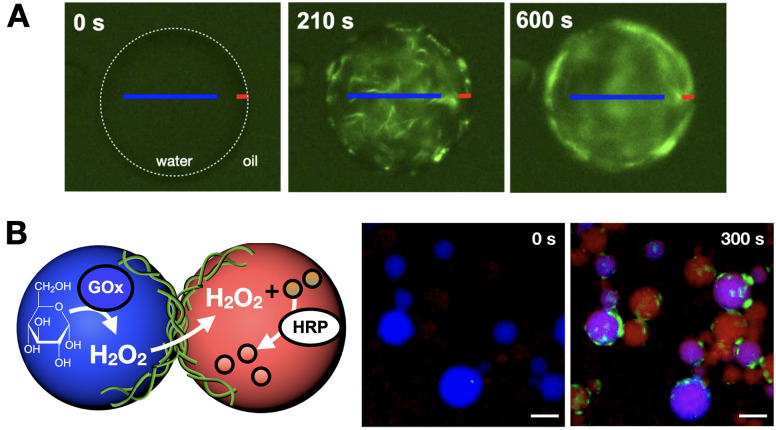
Confined synthesis and self-assembly of peptide amphiphiles to mimic cytoskeleton structure and function. (A) Fibres are first assembled throughout the droplet interior (0–210 s) to then accumulate at the interface over time (600 s), thus creating a fibrillar cortex. (B) Two-step enzymatic cascade across droplet populations mediated by content exchange *via* connected fibre cortices. Successful reactivity results in the production of a red reporter (300 s). GOx = glucose oxidase, HRP = horseradish peroxidase. Scale bars = 70 µm. Adapted with permission from ref. 134, copyright 2021, Springer Nature.

We have developed a peptide amphiphile monomer capable of fibrillar self-assembly,^133^ which can be confined in water-in-oil droplets to mimic cytoskeletal structure and function.^134^ In this work, *in situ* production of self-assembling amphiphiles within droplets could generate fibre networks that over time accumulate at the cortex of these compartments like a cytoskeletal shell (Fig. 11A). Because these monomers are highly anionic, fibrillation can promote the selective uptake of small molecules from the medium based on charge. Neutralisation of these anionic cytoskeletons induced droplet fusion, as fibre cortices stabilise inter-droplet contacts by charge repulsion. However, interfacing cytoskeletons could connect droplets without coalescence, thus maintaining droplet integrity while allowing content exchange, which could be exploited to trigger a two-step enzymatic cascade across droplet populations (Fig. 11B).

## 5. Summary of peptide designs

In this final section, we classify the examples discussed above based on their supramolecular morphology: either elongated nanofibres and nanotubes, lamellar nanosheets and porous multilayers, or spherical coacervates and vesicles. This summary correlates peptide sequence with supramolecular structure, rationalising nanomaterial fabrication based on monomer design (*e.g.* amino acid composition, peptide derivatisation, *etc.*). A table at the end of this section helps compare all peptide sequences, supramolecular structures, assembly conditions and emergent properties.

### 5.1. Nanofibres and nanotubes

These assemblies elongate in one dimension of space, usually relying on inter-backbone H-bonding to direct the linear propagation of peptide monomers. As shown in Table 1, most nanofibre and nanotube monomers form linear β-sheets, generally containing hydrophobic residues (*e.g.* Val, Ala, Leu, *etc.*) to promote lateral peptide association by steric zipping as cross-β interfaces (Fig. 1A).^135^ In many designs, charged residues (*e.g.* Glu, Lys, His) generate inter-monomer repulsion on the surface of the assembly to prevent aggregation,^34,85,134^ while other peptides incorporate both cationic and anionic segments for the electrostatic cross-linking of fibres.^45,127^ The attachment of fatty acids (*e.g.* lauric, palmitic, *etc.*) drives monomer association in water *via* non-directional hydrophobic packing, allowing fatty acid chains to cluster without interfering with the directional (*i.e.* linear) propagation of peptides by H-bonds.^74,95,125^ Interestingly, nanotubes can be obtained from very similar peptide sequences to those producing nanofibres,^41,45^ the former being usually assembled hierarchically from pre-organised structures over time (up to weeks).^136^ Despite all these rational molecular design concepts, fibrillar and tubular peptide assemblies are highly polymorphic, and their final structure strongly depends on kinetic^137^ and energetic parameters (*e.g.* annealing).^138^ As a result, prediction of the supramolecular nanostructure is not possible directly from the monomer sequence alone and also requires systematic screening of environmental conditions and incubation times.

**Table 1:** Summary of peptide sequences with emergent properties discussed in the previous sections. Unless indicated otherwise (*e.g.*^D^F), these are all l-amino acids. Abbreviations: n/s (not specified), Nap (naphthalene), Im (imidazole), Catec (catechol), Ar_1_ (1-(4-formylbenzyl)-4-phenylpyridin-1-ium), ^β^A (beta-alanine), ^Me^A (2-aminoisobutyric acid), Fmoc (9-fluorenylmethoxycarbonyl), Ac (acetyl), TBDMSO (*tert*-butyldimethylsilyloxy), Bpy (2,2'-bipyridine), Orn (ornithine), Nva (norvaline), GlcNAc (*N*-acetylglucosamine), PEG_2_ (diethyleneglycol), DNA (nucleic acid conjugate)

Peptide sequence	Nanostructure	Secondary fold	Emergent property	Assembly trigger	Assembly conc. (mM)	Ref.
Lauryl-VVAGX-NH_2_ (X = E, K, HH)	Nanofibres	β-sheet	Catalysis	Concentration	0.05	34
Palmitoyl-H-OH		n/s	Catalysis	Concentration	30	40
H-KLVFFAE-OH		β-sheet	Catalysis	Coacervation	0.01	45
Dodecyl = NO-acetyl-F_3_E_2_-OH		β-sheet	Catalysis	Concentration	1	74
Fmoc-FF-OH		β-sheet	Structural amplification	pH	1	6
H-P(γ-TBDMSO)^D^E(dodecyl)-dodecyl		n/s	Structural amplification	Temperature	10	83
Ac-F_3_K-NH_2_		β-sheet	Structural amplification	pH	8	85
Palmitoyl-A_4_K_4_GRADA-NH_2_		β-sheet	Structural amplification	pH	0.75	95
GlcNAc-(SG)_2_Q_2_K(FQ)_2_FEQ_2_-NH_2_		β-sheet	Structural amplification	Concentration	5	97
Ac-A_6_YD-OH		n/s	Life-like	Concentration	3	114
Palmitoyl-V_2_A_2_E_4_IKVAV-NH_2_		β-sheet	Life-like	Concentration	6	125
Palmitoyl-V_3_A_3_E_3_-NH_2_, H-E_3_A_3_V_3_K(lauryl)-NH_2_		β-sheet	Life-like	Concentration	10	126
Palmitoyl-V_3_A_3_E_3_K_3_-PEG_2_-DNA, palmitoyl-V_3_A_3_E_3_K_3_-NH_2_		β-sheet	Life-like	DNA hybridisation	0.5	127
Fmoc-FF-OH, Fmoc-FF-PEG_2_-DNA		β-sheet	Life-like	DNA hybridisation	2	132
Alkyl = NO-acetyl-V_2_A_2_E_2_-NH_2_		β-sheet	Life-like	Concentration	10	133
Octyl = NO-capryloyl-V_2_A_2_E_2_-NH_2_		β-sheet	Life-like	Concentration	1	134
Im-KLVFFAL-NH_2_	Nanotubes	β-sheet	Catalysis	Concentration	1	41
Nap-FFXH-OH (X = D, H, S, R, K)		β-sheet	Catalysis	pH	1.6–33	47
Ac-I_4_^D^KK-NH_2_		β-sheet	Structural amplification	Concentration	16	80
H_*n*_FF (*n* = 1–20)	Nanosheets	β-sheet	Catalysis	pH	n/s	49
cyclo(W^D^LH^D^EQ^D^HE^D^L)		β-sheet	Structural amplification	Concentration	0.1	98
cyclo(W^D^LE^D^HL^D^WL^D^EH^D^L)		β-sheet	Structural amplification	Concentration	0.1	100
Bpy-L^Me^AA^Me^AL^Me^AQ^Me^AL-Bpy	Porous multilayer	α-helix	Structural amplification	Temperature	12	90
Fmoc-P_4_-NH_2_		Helix	Structural amplification	Temperature	40	91
cyclo[(KLVFFAEOrn)_2_]		β-sheet	Structural amplification	Crystallisation	5	92
H-F^D^NvaF-OH		β-sheet	Structural amplification	Concentration	8	93
H-^D^F(4-fluoro)F-OH		β-sheet	Structural amplification	Concentration	38	94
H-KVYFSIPWRVPM-NH_2_	Coacervates	β-sheet	Catalysis	Ionic strength	0.66	44
Catec-SDLVFFH-NH_2_		β-sheet	Catalysis	Concentration	0.6	46
Ar_1_-^β^AFF^Me^A-NH_2_		n/s	Catalysis	Concentration	20	54
(H-FF)_2_-cystamine		n/s	Catalysis	pH, concentration	1.35	55
Oligo(C)		n/s	Catalysis	Concentration	1	72
H-FFM-OMe, H-MFF-OMe		n/s	Life-like	pH, concentration	2.4	117
Ac-FRGRGRGD-OH		n/s	Life-like	Electrostatic complexation	23	119
H-LVFFAR_9_-OH		β-sheet	Life-like	Electrostatic complexation	5	122
(VPGKG)_40_(VPGIG)_30_	Vesicles	n/s	Life-like	Concentration	0.001–0.05	111
[(VPGRG)_5_(VPGQG)_5_]_2_(VPGFG)_20_		Random coil	Life-like	Concentration	0.3	113

### 5.2. Nanosheets and porous multilayers

Engineering peptide self-assembly to produce lamellar structures requires fine control over the monomer's geometry to present binding motifs at precise angles. For example, peptides assembling into β-sheets present axial H-bonding contacts perpendicular to side chain binding surfaces (Fig. 1A),^49^ thus allowing the ordered propagation of peptides in these two dimensions. On this basis, enantiomerically pure peptides display consecutive side chains on alternating sides of the β-sheet, unlike peptides made of periodic D–L sequences, which segregate all side chains on one face of the β-sheet, allowing the assembly of porous materials (Fig. 1C)^93^ and hierarchical nanosheet structures (Fig. 8).^98,100^ Alternatively, helical peptide folds also allow the presentation of side chains at specific angles around the helical axis, as discussed here for the fabrication of porous supramolecular multilayers (Fig. 6A),^90,91^ and applicable to a wide variety of exohelical peptide topologies.^139^ Overall, understanding the geometry of secondary peptide structures is key to rationally designing lamellar assemblies, which serve as scaffolds for positioning the required amino acids at binding and non-binding domains.

### 5.3. Coacervates and vesicles

The supramolecular architectures discussed in Section 5.1. and 5.2. consist of highly ordered peptide lattices, where monomers self-assemble into defined molecular arrays with restricted conformation and mobility. In contrast, coacervates and vesicles are highly dynamic structures, where monomers can rapidly exchange with the solution and/or diffuse across the assembly. Peptide coacervates are membrane-less droplets formed by aggregation of disordered domains,^140^ which can be found in some of the monomers discussed above, like those containing non-peptidic aromatic substituents,^46,54^ flexible linkers,^55^ truncated sequences,^117^ or unfolded oligopeptides.^72^ Charged peptides can also undergo complex coacervation by disordered aggregation with oppositely charged macromolecules.^119,122^ As their behaviour and stability are determined by weak supramolecular interactions, coacervates can adapt their properties to environmental conditions that affect these bonds (*e.g.* temperature, pH, *etc.*). In this regard, peptide coacervates have attracted much attention as dynamic protocell models and life-like materials,^102,140^ which can accumulate and freely exchange substances with the surroundings without the permeability restrictions of a lipid membrane. Alternatively, peptide vesicles can be assembled from large amphiphilic peptide polymers,^111,113^ which create an impermeable boundary to control concentration gradients and molecular composition across their membrane.

## 6. Conclusions and outlook

This review puts in perspective the supramolecular cooperativity of peptide nanostructures, whereby assembled peptide monomers show emergent properties that transcend the sum of their individual features. We have shown functional responses emerging from collective monomer behaviour, such as catalysis, structural amplification, hierarchical assembly pathways and life-like function, amongst others. These phenomena are usually reviewed and discussed individually, as isolated topics with little connection between the fields and applications covered herein. We believe that integrating advances in monomer design, applications and mechanistic understanding into a single broader topic, here termed *emergent properties*, would benefit all these areas of research.

Our contributions to these areas have been highlighted at the end of each section, including autocatalytic systems, hierarchical self-assembling monomers and functional cytoskeleton mimics. We have studied a variety of peptide designs and supramolecular architectures that have contributed to advancing peptide nanotechnology, both conceptually and in application. Current efforts from our group are focusing on transferring supramolecular cooperativity and peptide self-assembly to biological systems, aiming to expand the design of new therapeutics from small single molecules to supramolecular nanostructures.

Based on the discussions included in this article, further development of cooperative peptide behaviour faces some interesting challenges ahead. To tackle the disconnect between fields of study, combined results from different applications and designs should be fed into machine learning platforms to identify structure-behaviour patterns and develop prediction models across areas of specialisation. In this regard, both monomer and supramolecular structure must be considered as inputs, as changes in assembly conditions can produce polymorphs with very distinct behaviour. Another strategic challenge is the application of these materials in biology, for example, catalytic and luminescent systems, where supramolecular stability in complex media and bio-orthogonality are required for function and compatibility. Protocellular systems could be an affordable and versatile alternative to certain cell therapies and transplants, and could replicate healthy cell function by integration of natural biochemical signalling for endocrine connection with the patient.

The fundamental and applied concepts developed in this field can have a strong impact on protein behaviour, biomolecular folding and function, responsive nanomaterial design and biomedical technologies, being directly linked to cell biology and biomaterial engineering. Supramolecular cooperativity sits at the very foundation of life, and the deconvolution of its basic principles and modes of action will surely resonate beyond chemistry, shedding light into the past to help explain an abiogenic origin of life, and into the future, towards developing new advanced materials with cooperating components.

## Conflicts of interest

There are no conflicts to declare.

## Data Availability

No primary research results, software or code have been included, and no new data were generated or analysed as part of this review.
